# Prognostic Impact of WT-1 Gene Expression in Egyptian Children with Acute Lymphoblastic Leukemia

**DOI:** 10.4084/MJHID.2016.008

**Published:** 2016-01-01

**Authors:** Adel A Hagag, Ibrahim M Badraia, Samir M Hassan, Amal E Abd El-Lateef

**Affiliations:** 1 Pediatrics Departments, Faculty of Medicine, Tanta University, Egypt; 2 Clinical Pathology Departments, Faculty of Medicine, Tanta University, Egypt

## Abstract

**Background:**

Acute lymphoblastic leukemia (ALL) is the most common childhood cancer representing 23% of pediatric cancers. Wilms’ tumor -1 gene is a novel prognostic factor, minimal residual disease marker and therapeutic target in acute leukemia.

**Aim of the work:**

The aim of this work was to study the impact of WT-1 gene expression in the prognosis of ALL.

**Patients and methods:**

This study was conducted on 40 Egyptian children with newly diagnosed ALL who were subjected to full history taking, thorough clinical examination and laboratory investigations including; complete blood count, LDH, BM aspiration, cytochemistry, immunophenotyping, FISH technique for detection of t(12;21) and t(9;22) and assessment of WT-1 Gene by real-time PCR in BM samples at time of diagnosis.

**Results:**

Positive WT-1 gene expression was found in 22 cases (55%) and negative expression in 18 cases (45%). Positive WT-1 gene expression group (n=22) includes 14 males and 8 females with mean age at presentation of 5.261 ± 0.811 while negative WT-1 gene expression group (n=18) includes 12 males and 6 females with mean age at diagnosis of 9.669 ± 3.731 with significantly older age in negative WT-1 gene expression group but no significant differences between positive and negative WT-1 gene expression groups regarding sex and clinical presentations. There were no significant differences in platelets and WBCs counts, hemoglobin and LDH levels and the number of peripheral blood and BM blast cells at diagnosis between positive and negative WT-1 gene expression groups but after induction therapy there were significantly lower BM blast cells in positive WT-1 gene expression group. There were no statistically significant differences between positive and negative WT-1 gene expression groups regarding immunophenotyping and chromosomal translocations including t(12;21) and t(9;22). There were a significantly higher relapse and death rate and a lower rate of CR, DFS, and OAS in negative WT-1 gene expression group. MRD at end of induction therapy was found in 14 cases out of 40 patients. There were significantly higher number of patients with MRD+ in negative WT-1 gene expression group (After the therapy 20 out of 22 (89%) patients with positive WT-1 gene expression attained a negative MRD, while only 6 out of 18 (33%) with negative WT-1 attained a negative MRD) (p-value = 0.006).

**Conclusions and Recommendation:**

WT-1 gene expression is an important prognostic factor in patients with ALL, being able to prognosticate a negative MRD. Therefore, we can recommend its incorporation into novel risk-adapted therapeutic strategies in patients with ALL.

## Introduction

Acute lymphoblastic leukemia (ALL) is the most common childhood cancer representing 23% of cancer diagnoses among children younger than 15 years. ALL occurs at an annual rate of approximately 30–40 per million. A sharp peak in ALL incidence is observed among children aged 2 – 3 years (>80 per million per year), with rates decreasing to 20 per million for ages 8–10 years.^1^

With improvements in diagnosis and treatment, overall cure rates for children with ALL have reached 90%. The use of risk-adapted treatment protocols has improved cure rates while limiting the toxicity of therapy.^2^ Among children with ALL, more than 95% attain remission and 75% – 85% survive free of leukemia recurrence at least 5 years from diagnosis with current treatments that incorporate systemic chemotherapy and specific central nervous system preventive therapy.^3^

Relapse is the leading cause of treatment failure for patients with ALL.^4^ Relapse originates from leukemic cells that are resistant to chemotherapy but become undetectable after initial treatment in most cases. Nevertheless, methods more sensitive than microscopic examination can demonstrate leukemic cells in a proportion of samples with no morphologic evidence of leukemia, a finding termed “minimal residual disease (MRD).^5^ MRD is currently the most powerful prognostic indicator in childhood ALL^6^ and has been introduced into many treatment protocols for risk assignment and selection of therapeutic regimens.^4^

WT1 gene has been identified in the developing kidney and in various malignancies.^7^ Real-time quantitative PCR (RQ-PCR) is an accurate method of quantifying WT1 expression. WT1 gene over-expression was demonstrated in diagnostic samples of many types of leukemia^8^ and this gene is considered to be an excellent tool for monitoring minimal residual disease in 70% of acute myeloid leukemia patients.^9^

The scope of the present work is to study the impact of WT-1 gene expression in children with acute lymphoblastic leukemia.

## Subjects and Methods

After approval from ethical committee of Tanta University research center and written consent from the parents of all children included in this study, the present study was conducted on 40 patients with newly diagnosed ALL who were admitted in Hematology Unit, Pediatric department, Tanta University Hospital in the period from January 2011 to January 2015 including 26 males and 14 females with their ages ranging from 4–15 years with mean age value of 7.24 ± 3.35 years. Patients included in this study were followed up clinically and by blast cells count in BM on day 21 of induction and for MRD in BM after induction and thereafter for 2 years to assess prognosis and were treated according to ALL-protocol of therapy.^10^–^14^

All patients were subjected to the following:

- Full history taking.
- Thorough clinical examination with particular attention to fever, pallor, purpura, hepatomegaly, splenomegaly, and lymphadenopathy.
- Laboratory investigations

### Specimen collection and handling

Three ml of venous blood were collected under complete aseptic technique. They were delivered into 2 tubes: 1 ml blood into a tube containing EDTA for complete blood count and 2 ml blood into a plain tube for assessment of Lactate dehydrogenase levels. Two ml of bone marrow aspirate were drawn into a sterile tube containing EDTA for mononuclear cell separation for polymerase chain reaction (PCR).

Laboratory investigations include the following:

Complete blood count.
Lactate dehydrogenase (LDH)

Bone marrow aspiration with cytochemical examination with Sudan black and Myeloperoxidase and immunophenotyping which was performed on gated blast cells from bone marrow samples by flow cytometry using an extensive panel of Fluorescin Isothiocyanate [FITC] and Phycoerythrin [PE] conjugated monoclonal antibodies [MoAbs] for diagnosis and subtyping of ALL including T-cell lymphoid markers (CD3, CD5, CD7), B-cell markers (CD10, CD19, CD22 and cy- immunoglobulin) and Myeloid cell markers (CD13, CD33).^15^

Fluorescent in situ hybridization (FISH) for detection of t(9;22) and t(12;21): BM specimens were cultured for 72 hours at 37ºC in RPMI medium supplemented with 10% fetal bovine serum without the addition of any mitogen (un-stimulated). Colcemid (0.02ug/ml) was added to the cultures 30 minutes before harvest. After 30 minutes of hypotonic treatment with 0.075M KCL, the cells were fixed with methanol and acetic acid (3:1) and cells were made into slide preparations. Hybridization mixture (10ul) was then applied to each slide, which was covering slipped and sealed. Hybridization solution contained (hybridization buffer, purified water, and a specific probe). Specific BCR-ABL dual-color DNA probe hybridizes to chromosome 9q^34^ and chromosome 22q^11.2^ to detect t(9;22)(q^34^,q^11.2^) and TEL-AML dual-color DNA probe hybridizes to chromosome 12p^13^and chromosome 21q^22^ to detect t(12;21)(p^13^,q^22^) were used. FISH assay was performed according to (Vysis, Abbott # 32-190022) manufacturer’s instructions. The analysis was done under a fluorescence microscope equipped with Quips spectra vision hard and software.^16^

### Assessment of WT-1 Gene by real-time PCR in bone marrow samples at the time of diagnosis

**Figure 1:**
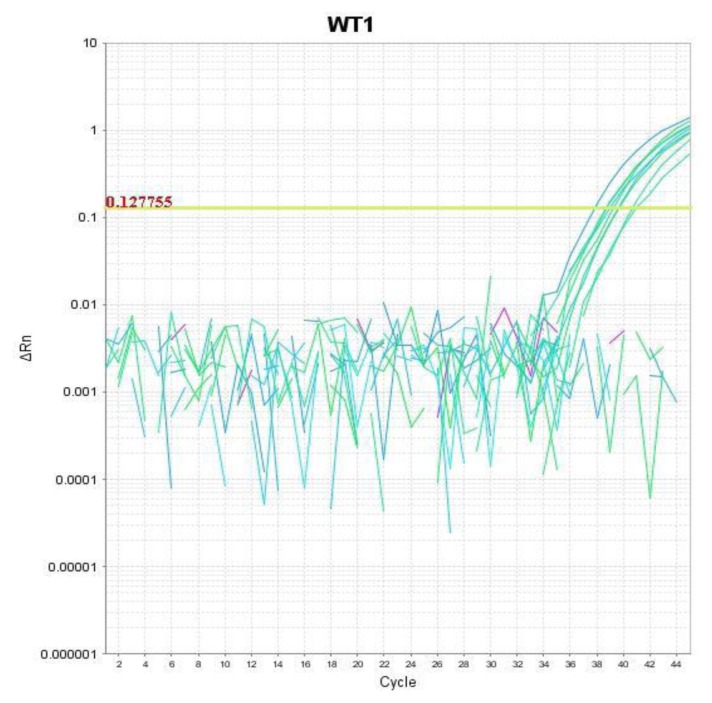
A case with positive expression of WT-1 gene by quantitative real-time PCR. Amplification Plot showing the log of the change in the fluorescence plotted versus cycle number (ΔRn vs. Cycle).

Mononuclear cells were separated from the aspirated bone marrow samples by centrifugation on Density gradient medium (Lymph Prep). RNA was isolated from the samples using an RNA easy Mini Kit according to the Qiagen Protocol (QIAGEN GmbH, Hilden, Germany) for isolation of total RNA and the amount of extracted total RNA was estimated quantitatively by spectrophotometric measurement and qualitatively by electrophoresis. Patients RNA was reverse transcripted using transcription first strand cDNA synthesis kit (ROCH diagnostic GmbH, Mannheim Germany) and then stored at −80ºC until PCR amplification. The cDNA is used as a template to amplify WT-1 gene and the cDNA normalized using glyceraldehyde -3 phosphate dehydrogenase as a housekeeping gene. The expression is calculated relative to Housekeeping gene, and so the value as a ratio to this gene is either expressed (positive WT-1 gene expression) or not expressed (negative WT-1 gene expression). WT1 expression in peripheral blood was examined from 10 healthy children as control, and the cut-off value is determined relative to controls. DNA amplification of the WT-1gene was done by real-time PCR using Gene Amp 5700 Sequence Detection System (Applied Biosystems). Using the primers and probe for WT1 (Applied Biosystems, Foster City, California, USA) and Standard, primers and probes of GAPDH (Applied Biosystems, Foster City, California, USA), the Light Cycler TaqMan Master protocol was followed according to manufacturer’s instructions (Figure 1). 17

### Real-time PCR, Primers, and Probes

WT1 was amplified using: *Forward WT-1 Gene primer:* 5′-GATAACCACACAACGCCCATC-3′,

### Reverse WT-1 Gene primer

5′-CACACGTCGCACATCCTGAAT-3′; with annealing temperature 56 °C.

### WT-1 Gene probe

5′-FAM-ACACCGTGCGTGTGTATTCTGTATTGG-TAMRA-3′ was designed using Primer-Express software (PE Biosystems, Foster city, CA, USA). Real-time PCR amplification and data analysis were performed using the ABI Prism 5700 Sequence Detector System (PM Biosystems). Reactions were performed using real-time PCR 7000 sequence detection system (Applied Biosystems, Foster city, CA, USA) (11).

### Detection of MRD by RT- PCR of antigen receptor gene rearrangements

This method aims to generate clone-specific primers with a sensitivity of at least 10^−4^. The first step involves identification and sequencing of clone-specific antigen receptor gene rearrangements. This step is followed by a primer design in which the clone-specific sequence is examined to identify potential allele-specific oligonucleotides. The specificity and sensitivity of these primers are then tested. Post-induction DNA is then subjected allele-specific priming and the results compared with those produced from dilution of leukemic DNA into BC DNA. Controlled for variation in amplifiability of individual DNA samples is achieved using a control PCR, in this case, β-globin.

Primers were purchased from a commercial supplier (Invitrogen, Carlsbad, CA). Direct sequencing of PCR product was done**.** Clonal PCR products were excised and purified using QIA quick gel extraction kits (QIAGEN, Valencia, CA). Purified PCR fragments were sequenced directly by the Dana-Farber/Harvard Cancer Center Core Sequencing Facility (Boston, MA). Sequence reactions were analyzed on an Applied Biosystems 3700 capillary sequencer using Big Dye Terminator Chemistry version 2 (Applied Biosystems, Foster City, CA). The relevant consensus forward and reverse primers were used as sequence primers to obtain the sequence of both strand**s** Nucleotide sequences were aligned using DNA star software (DNASTAR, Madison, WI). TCR gene segments were identified using the Immunogenetics Database (http://imgt.cines.fr, IMGT; European Bioinformatics Institute, Montpellier, France)**.**^18^

### Protocol of treatment utilized in the studied ALL patients at diagnosis:^10^–^14^

#### Induction (6 weeks)

IV Vincristine 1.5mg/kg/m^2^/week (days 0, 7, 14, 21, 28, 35), Doxorubicin 25mg/m^2^/week IV infusion (days 0, 7, 14, 21, 28, 35), asparaginase 6000 u/m^2^ SC on alternate days (10 doses) and oral prednisone 40mg/m^2^/day for 6 weeks. On day 21, BM aspiration was done; If BM blast cells is more than 5 %, we add etoposide 100 mg/m2/dose IV (days 22, 25, 29), cyclophosphamide 750 mg/m^2^/dose IV infusion (days 22, 25, 29), aracytin 100/m^2^/dose IV (days 22, 25, 29), and methotrexate (MTX) 5g/m^2^ over 4 hours on day 28.^10^

#### Consolidation (9 weeks)

IV methotrexate 1gm/m^2^/dose over 24 hour infusion on days 0, 21, 42 and 63, oral mercaptopurine 60 mg/m^2^ daily on days 0–13 and 28–41, IV vincristine 1.5 mg/m^2^ on days 14, 21, 42 and 49, PEG asparaginase 2,500 units/m^2^ IM on days 14 and 22, cyclophosphamide 750 mg/m^2^/dose IV infusion on days 0 and 28, aracytin 100/m^2^/dose IV on days 1–4, 8–11, 29–32 and 36–39 and age-adjusted intrathecal methotrexate on days 1,8,15 and 22.^11^, ^12^

#### Interim maintenance (6 weeks)

IV Vincristine 1.5 mg/m^2^ on days 0, 10, 20, 30, 40, IV methotrexate starting dose of 100 mg/m^2^/dose over 10–15 minutes on day 0 thereafter escalate by 50 mg/m^2^/dose on days 10, 20, 30 and 40, PEG asparaginase 2,500 units/m^2^ IM on days 1 and 21 and age-adjusted intrathecal methotrexate on days 0 and 30.^12^

#### Delayed–intensification (6 weeks)

Oral dexamethasone (10 mg/m^2^/day on days 1–7 and 14–21, IV vincristine 1.5 mg/m^2^ on days 0, 7 and 14, IM or IV pegylated L-asparaginase 2500 u/m^2^ on day 4, doxorubicin 25 mg/m^2^ IV on days 0, 7 and 14, IV cyclophosphamide 1gm/m^2^ over 30 minutes on day 28, oral 6-thioguanine 60 mg/m^2^ on days 28–41, aracytin 75mg/m^2^ on days 29–32 and 36–39 and age-adjusted intrathecal MTX on day 28.^13^

#### Maintenance (30 months)

Weekly IV methotrexate 20 mg/m^2^, prednisone 120 mg/m2/day for 5 days every 3 weeks, vincristine 2mg/m^2^ IV every 3 weeks, oral 6-mercaptopurine 50 mg/m^2^/day for 14 days every 3 weeks and age-adjusted intrathecal MTX every 18 weeks.^14^

#### Definition of disease response and relapse

Complete remission (CR) is defined as a cellularity of more than 20% with fewer than 5% blasts in BM after induction chemotherapy. Relapse is defined by appearance of more than 50% lymphoblasts in a single BM aspirate or more than 25% lymphoblasts of two or more BM aspirate and 2% or more circulating lymphoblasts or progressive repopulation of lymphoblasts in excess of 5% culminating in more than 25% of two or more BM samples separated by 1 week or more or leukemic cell infiltration in extramedullary organs as gonads or lymphoblasts in CSF with cell count greater than 5 WBCs/mm^3^.^13^

### Statistical analysis

The collected data were organized, tabulated and statistically analyzed using SPSS version 13. All Data expressed in terms of mean values ± SD. The difference between two means was statistically analyzed using the student (t) test. Chi-square test (X^2^) and Fischer exact test were used as a test of significance. For comparison between means of two different groups, parametric analysis (t-test) and non-parametric analysis (Mann- Whitney U test) were used. Significance was adapted at P<0.05. Log-rank test were used to assess survival.^19^

## Results

Positive WT-1 gene expression was found in 22 cases (55%) and negative expression in 18 cases (45%).

Positive WT-1 gene expression group (n=22) includes 14 males and 8 females with mean age at diagnosis of 5.261 ± 0.811 in whom pallor was found in 22 cases, purpura in 22 cases, splenomegaly in 6 cases, hepatomegaly in 8 cases, lymphadenopathy in 8 cases and bone-ache in 2 cases while negative WT-1 gene expression group (n=18) includes 12 males and 6 females with mean age at diagnosis of 9.669 ± 3.731 in whom pallor was found in 10 cases, purpura in 18 cases, splenomegaly in 18 cases, hepatomegaly in 18 cases, lymphadenopathy in 12 cases and bone-ache in 8 cases with significantly older age at diagnosis in negative WT-1 gene expression group but no significant differences between positive and negative WT-1 gene expression groups regarding sex and clinical presentations (Table 1).

There were no significant differences in platelets and WBCs counts, Hb and LDH levels and number of peripheral blood and BM blast cells at diagnosis between positive and negative WT-1 gene expression groups but after induction therapy there were significantly lower BM blast cells in positive WT-1 gene expression group (Mean platelets count in positive WT-1 gene expression group was 25.11 ± 9.07 versus 48.09 ±37.65 in negative group with p-value of 0.09, mean WBCs count in positive WT-1 gene expression group was 26.25 ±18.23 versus 23.47 ±13.37 in negative group with p-value of 0.71, mean Hb level in positive WT-1 gene expression group was 8.39 ± 1.02 versus 9.39 ± 1.38 in negative group with p-value of 0.08, mean LDH level in positive WT-1 gene expression group was 1122.23± 277.16 versus 1089.83±271.66 in negative group with p-value of 0.099, mean peripheral blood blast cells at diagnosis in positive WT-1 gene expression group was 36.22 ±10.11 versus 33.52±16.43 in negative group with p-value of 0.075, mean BM blast cells at diagnosis in positive WT-1 gene expression group was 88.72±8.73 versus 81.55±7.55 in negative group with p-value of 0.068 and mean BM blast cells after induction in positive WT-1 gene group was 4.272±2.323 versus 7.577±2.988 in negative group with p-value of 0.0122 (Table 1).

There were no significant differences between positive and negative WT-1 gene expression groups regarding immunophenotyping and chromosomal translocations) (Positive WT-1 gene expression group (n=22) includes 7 cases with early pre-B, 13 cases with pre-B, 2 cases with T-cell ALL and 5 cases having t(12;21) and 2 cases having t(9;22) while negative WT-1 gene expression (n=18) includes 5 cases with early pre-B, 9 cases with pre-B and 4 cases with T-cell ALL and 4 cases having t(12;21) and 1 case with t(9;22) (Table 1).

There were significantly higher rates of relapse and death and lower rate of CR, DFS and OAS in negative WT-1 gene expression group compared with positive group (in 22 cases with positive WT-1 gene expression; 19 achieved complete remission, 2 cases relapsed and 1 case died while in 18 cases with negative WT-1 gene expression; 6 achieved complete remission, 8 cases relapsed and 4 cases died with median DFS in positive WT-1 gene expression group of 23.52 months compared with 13.71 months in negative WT-1 gene expression group and median OAS in positive WT-1 gene expression group of 23.95 months compared with 16.8 months in negative WT-1 gene expression group). (Table 2 and 5).

Minimal residual disease at the end of induction therapy was found in 14 out of 40 patients. There was a significantly higher number of cases with MRD+ in negative WT-1 gene expression group (After the induction therapy 20 out of 22 (89%) patients with positive WT-1 gene expression attained a negative MRD, while only 6 out of 18 (33%) with negative WT-1 attained a negative MRD) (p-value = 0.006) (Table 4). MRD at the end of induction was associated with lower remission and higher relapse rates compared with MRD negativity (p-value = 0.026) (In total number of 26 cases with negative MRD, 21 cases achieved and maintained CR, 3 cases died and 2 case relapsed while in 14 cases with positive MRD, only 4 cases achieved and maintained CR, 8 cases relapsed and 2 cases died). (Table 3).

## Discussion

ALL is the most common childhood malignancy representing nearly one-third of all pediatric cancers**.**^20^ WT-1 gene is a tumor suppressor gene extracted initially from cells of Wilms’ tumor. The gene encodes cysteine-histidine zinc finger, a transcription factor which interacts with multiple hematopoiesis regulation factors, regulates the transcription and expression of genes, as well as takes part in the proliferation, apoptosis, and differentiation of hematopoietic cells.^21^ As a transcription factors, the product encoded by WT-1 gene can activate or inhibit the proliferation of cells, and expressions of differentiation and regulation genes.^22^

Several studies indicated that WT1 overexpression is an independent risk factor for relapse in acute leukemia**.**^23^ However, in childhood ALL, abnormally low as well as abnormally high WT1 levels were shown to be associated with poor outcome**.**^24^ The frequently observed WT1 overexpression in acute leukemia renders it an attractive marker for minimal residual disease assays.^25^

The present study was designed to use RT- PCR in studying the impact of WT-1 gene expression in 40 Egyptian children with ALL.

In this study positive WT-1 gene expression was detected in 55% and negative WT-1 expression in 45% of studied patients. This datum is not in agreement with Sadek et al. 2011,^26^ who found WT1 overexpression in 89% of pediatric ALL patients and Ibrahim et al. 2015^27^ who found WT1 expression at diagnosis in 14 % of ALL patients.

In our study, older children presented at the diagnosis more frequently a negative WT-1 gene expression. This datum seems to be not in agreement with, Boublikova et al. 2006^24^ who found that; children ≥ 10 years and ≤ 1 year old had significantly higher WT1 expression than children between 1–10 years of age. However, the age ranges of our two groups were different and difficult to compare.

In the present study, there were no significant differences in WT-1 gene expression between males and females. This is in agreement with Liu et al. 2014^21^ who studied WT1 gene expression by RT- PCR in the BM of 228 patients with hematologic neoplasms (leukemia, lymphoma, and multiple myeloma) and found no significant difference in WT1 expression levels between males and females but this is not in agreement with Sadek et al. 2011,^26^ who found significantly higher WT1-gene expression in males.

In the present work, there were no significant differences between positive and negative WT-1 gene expression groups as regard clinical presentations and laboratory data at the time of diagnosis. All this is in agreement with Sadek et al. 2011(26) and Ibrahim et al. 2015^27^ who found no significant differences between positive and negative WT-1 gene expression regarding clinical presentations and laboratory investigations including WBCs, platelets, blast cells counts and Hb level, immunophenotyping, and percentage of BM blasts.

In the present study; there were a significantly higher remission and lower relapse rates in positive WT-1 gene expression group that parallels with an increased proportion of children obtaining a negative MRD after therapy. However, children with positive WT1 at diagnosis were also younger. The major response to therapy of children positive to WT1 could be related due to the fact that WT-1 gene is a tumor suppressor gene which interacts with multiple hematopoiesis regulation factors, regulates the transcription and expression of genes, as well as takes part in the proliferation, apoptosis, and differentiation of hematopoietic cells.^21^,^22^ Thus Wilms’ tumor-1 protein (WT1) is a transcription factor that can either activate or repress genes to regulate cell growth, apoptosis and differentiation. WT1 can act as either a tumor suppressor or an oncogene.^22^

Accordingly, Boublikova et al. 2006^24^ found a higher risk of relapse in lymphoid leukemic children with WT-1 gene either overexpressed or underexpressed. The increased rate of relapse was more pronounced in children with abnormally low WT-1 gene expression, and may be explained by the tumor suppressor effect of WT1 gene. The relapse in children with abnormally high WT1 was associated with older age, over ten years, or age below one year, both conditions are well known to be bad prognosticators. The quantitative assessment of the WT1 gene transcript has been utilized as a marker of MRD in AML after induction and consolidation^7^,^9^ and can be a predictor of relapse.^23^ Data of WT1 as a marker of the presence of MRD are scarce and contradictory. Zhang et al. 2015^28^ studied WT1 expression levels in BM samples from 107 children with ALL and 35 children with AML at diagnosis, after induction, and consolidation therapy. He found that WT1 gene is a useful marker to predict relapse in childhood AML, through monitoring of MRD, but it is unreliable to predict relapse in ALL. Effectively he did not find any statistical difference between the levels of WT1 in ALL at diagnosis and after chemotherapy. So, an optimal value of cut-off was not found in ALL at variance with in childhood AML.

## Conclusion and Recommendation

The variation between this study and other previously published studies may be explained by different age groups, times and places of research. However, it is important to note, accordingly to Boublikova et al. 2006,^24^ that WT1 expression in childhood ALL is very variable and much lower than in AML or adult ALL. Therefore, WT1, will not be a useful marker for MRD detection in childhood ALL, however, it does represent a potential independent risk factor in childhood ALL. But its level could have a different significance. Interestingly, Boublikova et al.^24^ report a proportion of childhood ALL patients expressing WT1 at reduced levels at higher risk of relapse. In our hands, the high level of WT1 at the onset of disease was a predictor of a good response to therapy paralleling with a negative MDR after therapy. The small number of patients in our study and the heterogeneity in age, presentation, immunophenotype of studied patients, and the intrinsic variable expression of WT1 in ALL can justify the contradictory results. More studies need. However, the assessment of WT1 at the onset of the disease, associated with MDR testing after therapy, could improve the risk evaluation and then the novel risk-adapted therapeutic strategies in patients with ALL.

## Figures and Tables

**Table 1:** Comparison between positive and negative WT-1 gene expression groups regarding clinical and laboratory data at time of diagnosis.

Parameters		Positive WT-1 gene expression group (n=22)		Negative WT-1 gene expression group (n=18)		t test or X^2^	P- value

		N	%	N	%		

**Sex**	**Males**	14	35%	12	30%	0.020	0.88

	**Females**	8	20%	6	15%		

**Age**							
**Range (years)**		4–6		6–15		3.831	0.001*
**Mean**± **SD (years)**		(5.261 ± 0.811)		(9.669 ± 3.731)			

**Fever**	Present	14	35%	8	20%	5.45	0.06

**Pallor**	Present	22	55%	10	25%	3.64	0.05

**Purpura**	Present	14	35%	18	45%	2.13	0.144

**Lymphadenopathy**	Present	8	20%	12	30%	1.84	0.174

**Hepatomegaly**	Present	20	50%	18	45%	6.23	0.08

**Splenomegaly**	Present	19	47.5%	18	45%	14.03	0.075

**Bone ache**	Present	2	5%	8	20%	3.42	0.064

**Hb (gm/dl)**	Range	7.1 – 9.8		7 – 11		1.87	0.08
	Mean ±SD	8.39 ± 1.02		9.39 ± 1.38			

**Platelets (×10^3^/mm^3^)**	Range	13–101		14–37		1.78	0.09
	Mean ±SD	25.11 ± 9.07		48.09 ±37.65			

**TLC (×10^3^/mm^3^)**	Range	3.5–48		6.5–38		0.38	0.71
	Mean ±SD	26.25 ±18.23		23.47 ±13.37			

**Blast cells (%) in peripheral blood**	Range	7–55		9–62		0.862	0.075
	Mean ±SD	36.22 ±10.11		33.52±16.43			

**BM blast cells (%) before induction**	Range	75–100		72–95		1.939	0.068
	Mean ±SD	88.72±8.73		81.55±7.55			

**BM blast cells (%) after induction**	Range	2.5–11		4.5–12		2.785	0.0122*
	Mean ±SD	4.272±2.323		7.577±2.988			

**LDH (U/L)**	Range	760–1688		767–1715		0.79	0.099
	Mean ±SD	1122.23± 277.16		1089.83±271.66			

**Immunophenotyping**							
**Early Pre-B**	12 (30%)	7(17.5%)		5(12.5%)			
**Pre-B**	22 (55%)	13(32.5%)		9(22.5%)			
**T cell**	6 (15%)	2 (5%)		4(10%)		0.036	0.850

**Chromosomal translocation**							
**t(12;21)**	9	5(22.72)		4(22.22)			
**t(9;22)**	2	2(4.5%)		1(5%)		2.3	0.92

**WT-1 gene**	Range	0.166–9.1		0.001–0.165			
	Mean ±SD	4.52±2.53		0.023±0.056		3.4	0.023

* Significant (p<0.05).

SD=Standard deviation. TLC= Total leucocytic count. BM=bone marrow. LDH=lactate dehydrogenase.

**Table 2:** Outcome of studied patients in relation to WT-1 gene expression.

WT-1 gene expression		Patients outcome ( No=40)		
		Death (5 cases)	Remission (25 cases)	Relapse (10 cases)
**Positive WT-1 gene expression group (No=22) (55%)**		1 (2.5%)	19 (47.5%)	2(5%)
**Negative WT-1 gene expression group (No=18) (45%)**		4 (10%)	6 (15%)	8(20%)
**Total Number = 40 (100%)**		5 (12.5%)	25 (62.5%)	10 (25%)
**Chi-square**	X^2^	8.476		
	P-value	0.014^*^		

**Table 3:** Outcome of studied patients in relation to Minimal residual disease.

Minimal residual disease		Patients outcome ( No=40)		
		Death (5 cases)	Remission (25 cases)	Relapse (10 cases)
**Positive Minimal residual disease group (No=14) (35 %)**		2 (5%)	4 (10%)	8(20%)
**Negative Minimal residual disease group (No=26) (65 %)**		3 (7.5%)	21 (52.2%)	2 (5%)
**Total Number = 40 (100%)**		5 (12.5%)	25 (62.5%)	10 (25%)
**Chi-square**	X^2^	6.42		
	P-value	0.026*		

* Significant.

**Table 4:** Relation between WT-1 gene expression and MRD.

Minimal residual disease		WT-1 gene expression	
		Positive WT-1 expression (no=22) (55%)	Negative WT-1 expression (no=18) (45%)
**Positive Minimal residual disease group (no=14) (35 %)**		2 (5%) (11%)	12 (30%) (66%)
**Negative Minimal residual disease group (no=26) (65 %)**		20 (50 %) (89%)	6 (15 %) (34%)
**Chi-square**	X^2^	7.542	
	P-value	0.006*	

* Significant.

**Table 5:** Log Rank test of overall and disease-free survival.

	Overall survival			Log Rank	
	Median	SE	CI 95%	test value	P-value
**Positive**	23.63	0.860	17–26	1.490	0.045*
**Negative**	16.72	2.160	2–24		
	**Disease free survival**			**Log Rank**	
	**Median**	**SE**	**CI 95%**	**test value**	**P-value**
**Positive**	23.52	1.210	17–21	5.450	0.030*
**Negative**	13.71	0.890	5–12		

* Significant.

SE=Standard error. CI= Confidence Interval.
